# Investigating the Crystallization of a Hydroxycarbonate Precursor for a Ni–Mg–Catalyst through *In Situ* and *Ex Situ* Methods

**DOI:** 10.1021/acs.inorgchem.6c02129

**Published:** 2026-07-23

**Authors:** Anna Wolf, Sebastian Mangelsen, Malte Behrens

**Affiliations:** 9179 Institute of Inorganic Chemistry and Kiel Nano Surface and Interface Science (KiNSIS) Kiel University, Max-Eyth-Str. 2, 24118 Kiel, Germany

## Abstract

In this study, the crystallization of a coprecipitated precursor for a Ni catalyst suitable for green methanation is investigated. The catalyst is derived from a basic hydroxycarbonate where Ni^2+^ is partially replaced by Mg^2+^-cations forming a mixed solid-solution crystal of the composition (Ni_*1‑y*_Mg_*y*_)_12_(CO_3_)_8_(OH)_6_O·*m*H_2_O (with *y =* 0–0.5). The hydrothermal aging was identified as a key step in its synthesis, which previously remained unexplored. *Ex situ* PXRD, IR, EDX, SEM, and ICP-OES as well as *in situ* IR and pressure monitoring were used to study this reaction. In this aging step, the amorphous precipitate begins to transform into a crystalline solid-solution compound within 30 min after reaching hydrothermal conditions. The initial amorphous precipitate was examined, and its sum formula was established as (Ni_1–*x*_Mg_*x*_)_2_(CO_3_)­(OH)_2_·2.5H_2_O. The multimethod approach clarified the metal ion ratio evolution and the crystallization and carbonate incorporation as a function of time as the two hydroxycarbonate phases interconverted, enhancing understanding of the aqueous precursor chemistry and catalyst synthesis and showing the importance of dissolved species in the mother liquor in this reaction.

## Graphical abstract

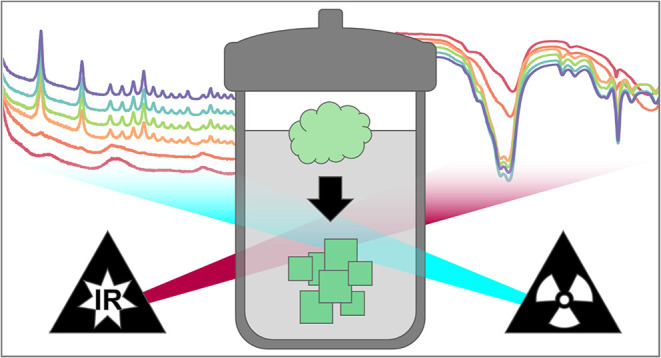

## Introduction

Solid catalysts are the backbone of the chemical industry. Beyond chemical production, heterogeneous catalysis can also create sustainable energy systems to address the accelerating issue of climate change. Particular attention must be paid to energy efficiency, environmental impact and economic profitability of existing and new processes in this context. The Power-to-X concept is hereby proposed to improve secure on-demand access to energy by creating solutions for energy storage, utilization and conversion.^1^ Since this is a large scale aim the expectations for catalysts in this field are high and the standards challenging. The catalysts need to match the demands of a cost-efficient material, preferably non noble metal, that excels in high activity, selectivity, durability and is able to accommodate feedstock flexibility.^2^ Moreover, it is a major selling point and accelerates implementation if the process or products can be integrated into an existing infrastructure.

We have recently introduced a new synthetic route to a non-noble nickel/magnesium oxide-based catalyst used in CO_2_ methanation that shows potential to fulfill these goals.^3^ This catalyst has been synthesized by coprecipitation and crystallization of a novel precursor compound and has been shown to outperform an industrial type benchmark catalyst at low temperatures below 300 °C, which is thermodynamically favored as well.^3,4^ The methanation of CO_2_ with green hydrogen for feeding it into the existing natural gas grid has a technology readiness level (TRL) of around 8 at present, with commercial test plants already running.^5^ Even though each catalyst is tailored to its specific use, Ni presents itself as highly versatile and the new catalyst can potentially assist a variety of different reactions including dry and/or steam reforming of methane, ammonia decomposition and electro catalysis.^1,4^ Aside of general issues of Ni such as the ethical side of nickel mining and the environmental side of pollution and toxicity,^6^ frequent practical complications include sintering and coke formation. Here, the support can have a major influence on the catalyzed reaction as well. Catalysts derived from (Ni,Mg)O solid solutions are of particular importance in the field.^7^ During catalytic methanation of carbon dioxide with hydrogen, the Mg in the form of MgO or (Ni,Mg)­O acts both as a support and, due to the basicity of the surface, as a CO_2_-adsorbing promoter. In these cases, the synthesis method (e.g., impregnation, coprecipitation, microemulsion, or sol–gel synthesis) is known to have a decisive influence on particle size and surface characteristics, which impacts the catalytic activity and selectivity. A recent study by Lai et al. on active sites in solid solution NiO-MgO catalysts provides evidence of Ni^δ+^ sites in a catalyst for dry reforming of methane, which could possibly also exist during methanation and, among other things, might contribute to the excellent activity.^8^

When originating from a solid solution (Ni_*1‑x*_Mg_*x*_O) the Mg to Ni ratio influences the reducibility of Ni and therefore particle size of the active metallic Ni particles. As investigated by Hu,^9^ the reducibility of Ni becomes increasingly harder with decreasing Ni fraction in the solid. The reason for that cannot be found in the M–O bonding strength since they are very much alike for Ni (*E*_Ni–O_ = 382 kJ/mol^10^) and Mg (*E*_Mg–O_ = 363 kJ/mol^10^). Since the energies required to break the H_2_ bond and form water during reduction would be the same for both metals, these can be disregarded. The difference therefore lies in the strength of the newly formed M–M bond. At 9 kJ/mol,^10^ this is significantly lower for Mg–Mg than for Ni–Ni at 200 kJ/mol.^10^ The formation of M–M bonds is therefore the driving force behind the reduction. As a result, a Ni atom that is surrounded exclusively by Mg atoms in the solid solution oxide will not be able to be reduced and thus remains in the solid solution.^9^ However, higher temperatures promote diffusion, allowing a higher degree of reduction to be achieved in the sample. For this reason, the proportion and distribution of different types of atoms influences the particle size of metallic Ni in the active catalyst. When there are a large number of Ni atoms, they are more likely to be in close proximity to each other, facilitating the formation of metallic bonds. Hereby the peak reduction temperature shifts to lower temperatures, making it easier to form larger domains. The dispersion at an atomic level—and thus the tendency to form smaller and more active particles—is best achieved with a single source precursor. Since this is only possible for an oxide solid solution at very high temperatures, the indirect route of coprecipitation followed by crystallization and subsequent calcination must be taken. The catalyst under study here originates from a coprecipitated bimetallic precursor and its structural and textural evolution towards a Ni/(Ni_1*‑x*_Mg_*x*_O) catalyst has been reported recently by Wolf et al.^3^ This study now investigates the formation of the crystalline product in the hydrothermal aging step regarding chemical phenomena in greater detail.

Our newly established synthesis requires a hydrothermal crystallization step subsequent to the coprecipitation to yield the novel precursor (Figure 1), that is a hydroxycarbonate with the sum formula (Ni_1‑*y*_Mg_*y*_)_12_(CO_3_)_8_(OH)_6_O·*m*H_2_O (with *y* *=* 0–0.5),^3^ which can be calcined to form the precatalyst solid solution (Ni_1‑*y*_Mg_*y*_O). Its crystal structure has been elucidated by Rincke et al.^11^ Using the coprecipitation approach, Ni^2+^ ions can be exchanged by Mg^2+^ cations in the precursor, resulting in a mixed crystal. To elucidate the chemical processes during hydrothermal aging, time-resolved sample series were taken and characterized *ex situ*, and an *in situ* IR study was performed to open up the “black box” of autoclave synthesis, which determines the crystallinity and composition of the precursor, the resulting solid solution and finally the metal–support interaction in the active catalyst.

**Figure 1:**
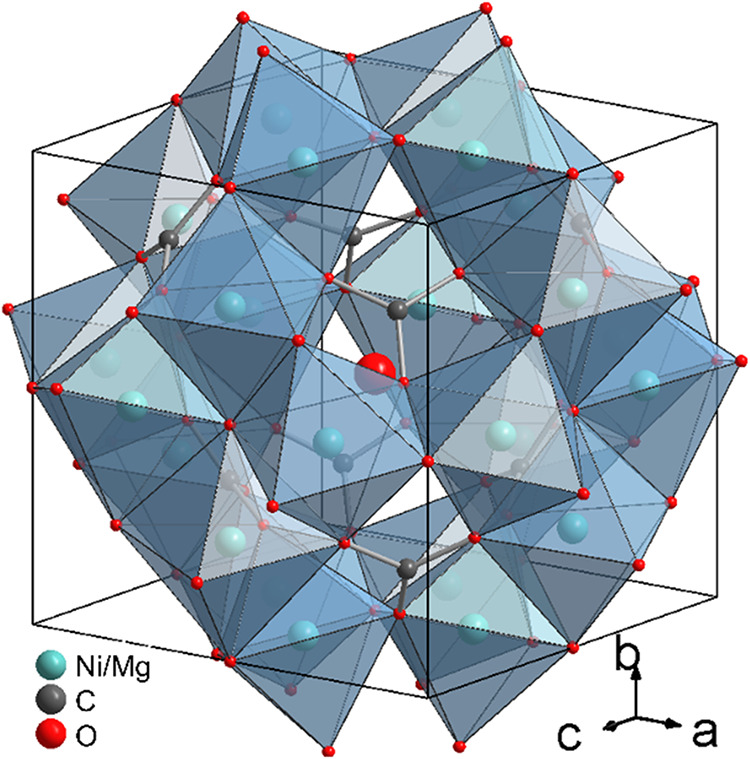
Crystal structure of the catalyst’s precursor (Ni_1–*y*_Mg_*y*_)_12_(CO_3_)_8_(OH)_6_O·*m*H_2_O.

## Experimental Section

### Characterization

#### PXRD

The patterns were recorded with a laboratory powder diffractometer Stadi P by STOE equipped with Mo–Kα_1_ radiation, Johann-type Ge (111) monochromator and a Mythen 1 K detector (Dectris) in transmission geometry. The samples were prepared as flat plates between to sheets of Scotch® brand adhesive tape.

TOPAS Academic version 6.0 was used for carrying out the structure refinements. Volume weighted domain sizes were calculated by the included routine as well.^11^

#### Elemental Analysis

The determination of the content and ratio of alkali, alkaline earth, and transition metals was conducted with an ICP-OES Avio 200 by PerkinElmer. For analysis of the elements C, H, N, and S, a Vario MICRO cube (Elementar) elemental analyzer was used.

#### Infrared Spectroscopy

The *ex situ* IR spectra were recorded on a Bruker ALPHA-P ATR MIR spectrometer in the range of 4000–500 cm^–1^. The *in situ* IR spectra were recorded with a ReactIR spectrometer by Mettler Toledo and AgX FiberConduit probe with a diamond tip with a resolution of 2 min. The region of 2000–650 cm^–1^ is covered.

#### SEM-EDX

For microscopy a Hitachi SU8700 scanning electron microscope with an Oxford EDX-Detector Ultim Max 100 was used. The EDX mappings were recorded with a beam voltage of 10 kV and at 10 Pa to minimize static charge effects while the high-resolution SEM images were collected at 3 kV and 10^–3^ Pa.

### Materials

For coprecipitation and analysis the following materials were used without further purification: Nickel nitrate hexahydrate (abcr, 99.9%), magnesium nitrate hexahydrate (Merck, 99%), sodium carbonate anhydrate (Grüssing, 99.5%), and nitric acid 65% (Honeywell, p.a.).

### Synthesis

The precursor syntheses were carried out in the automated laboratory reactor system EasyMax 102 (up to 100 mL) by Mettler Toledo. It is equipped with a glass reactor embedded in a solid-state thermostat. The parameters such as temperature, stirring speed, and pH were adjusted and monitored by a computer controlled regulatory cycle. A 1 M acidic metal salt solution with the desired Ni:Mg ratio was gravimetrically dosed into the prefilled reactor (50 mL of demin. water). The precipitating agent (1.6 M Na_2_CO_3_ solution) was dosed simultaneously by a membrane pump as well over 30 min. This way the pH was kept constant at a value of 9 at a temperature of 5 °C. The stirring speed was kept constant at 300 rpm. For the aging respectively crystallization, the slurry was transferred to a sealed vessel that is pressure-resistant and heated up to 100 °C. The previously published synthesis protocol the aging time was 20 h in a Teflon lined steel autoclave.^3^ In the study presented here the reactor type and the aging time vary.

## Results and Discussion

The coprecipitation of Ni and Mg into a single hydroxycarbonate phase can be challenging as the synthesis conditions need to allow for the precipitation of both metals, which occurs at different pH values, and for the correct concentration of carbonate and hydroxide anions, also depending on the pH, the carbonate equilibrium and the temperature. We have recently reported that the highly promising catalyst precursor (Ni_1‑*y*_Mg_*y*_)_12_(CO_3_)_8_(OH)_6_O·*m*H_2_O (with *y =* 0–0.5) can be synthesized by a combination of (co)­precipitation at constant pH of 9 and subsequent hydrothermal crystallization of the whole batch including the precipitate and the mother liquor.^3^ It was anticipated that Mg was not completely precipitated but incorporated in the solid over time during the hydrothermal treatment.^3^ For a deeper understanding of the formation of the precursor a time-resolved experiment was conducted: 6 Pyrex reaction tubes (10 mL) were filled with an equal amount (approximately 7 mL) of slurry from a 100 mL batch (for precipitation protocol see Figure S1) directly after (co)­precipitation of the Ni and Mg nitrate solution (molar metal ratio 7:3) with sodium carbonate solution. They were tightly sealed with a screw cap afterward and placed in a 103 °C aluminum heating block (without the possibility of stirring) creating hydrothermal conditions. The hydrothermal state is not reached due to heating above the solvents boiling point alone but rather by the decreased solubility of CO_2_ at elevated temperatures, vide infra. Those conditions are indeed necessary because a foregoing experiment in an open vessel yielded no crystalline product likely due to the loss of CO_2_ via the gas phase. Test tubes were taken from the heat at regular intervals of 15 min up to 1 h and at 2 h and immediately quenched in an ice bath, washed and dried.

The resulting bright green powders were characterized *ex situ* with ICP-OES and powder diffraction to examine the evolution of the elemental composition of the whole solid and of the phase composition and structure of the crystalline fraction, respectively. By the results of ICP-OES analysis, the Mg content in the solid was low directly after (co)­precipitation and approaches the target value of 30% in a manner of a logarithmic function over the course of 2 h (Figure 2a). After this time, the concentration of Mg in the solid did not reach the desired amount, but lingers at 27.8%. However, with longer aging times of 20 h Mg reaches the set metal ratio as is has been shown before.^3^ The powder diffraction patterns show a sudden rise in crystallinity at 30 min with the sample exhibiting the characteristic reflections for the target structure (Figure 2b). Before that, the patterns exhibit very broad yet distinct reflections, indicating reasonable short-range order. In the pattern of the sample collected after 15 min the reflections are somewhat more defined compared to the state at 0 min and they coincide to some extend with the positions of the subsequently crystallizing phase. The patterns of the samples collected after 30 min and longer aging time were suitable for Rietveld refinement. The results show a steadily rising lattice parameter *a* of the cubic structure with increasing Mg content (Figure 2c) compared to the one of the pure Ni hydroxy carbonate (8.3815 Å).^11^ This is indicated by a shift of reflections to smaller diffraction angles as the crystal ionic radius of Mg^2+^ is slightly larger than of Ni^2+^.^12^ The single metal site in the cubic crystal structure was calculated to be occupied by Mg by approximately 15(1)% for the 30 min sample up to 21(1)% for the 120 min sample. This is systematically lower compared to the values determined by ICP-OES and EDX, but in an acceptable range given the limitations of XRPD in this respect. When fixing the occupancy to the value determined by ICP-OES, the change in weighted-profile R-factor (r_wp_) is only miniscule. Still the data reproduces the trend in elemental composition, underscoring that the change in composition takes place in the crystalline material and is not or only to a limited extend rooted in amorphous fractions of the sample. The average domain size, which was calculated from the line profiles as a result of the refinement, grows over time from 3.5(2) nm after 30 min to 4.2(2) nm after 120 min of aging time as anticipated and the material remains in the nanoscaled regime. All difference plots of the refinements and their results presented in tabular form can be found in the Supporting Information (Figure S2 and Table S1). These results demonstrate that it is highly important to transfer the whole slurry to hydrothermal conditions because large proportions of Mg^2+^ cations are still in solution after the precipitation step.

**Figure 2:**
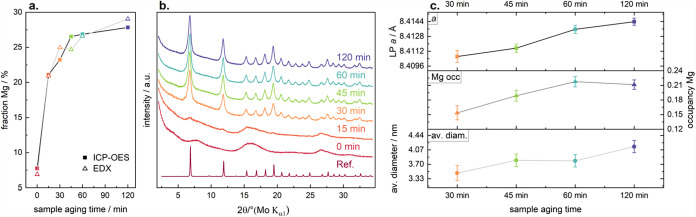
(a) Fraction of Mg regarding metal atoms determined by ICP-OES and EDX, (b) PXRD data of samples collected after times as indicated compared to a calculated pattern^11^ of Ni_12_(CO_3_)_8_(OH)_6_O. (c) Results of Rietveld refinements: lattice parameter (LP) *a* of the cubic precursor structure (top), occupancy of Mg in the metal site and average diameter of the crystallite domains (bottom).

We now turn to the analysis of the IR spectra of the samples from this time series. Spectra from the literature were consulted to identify the observed initial X-ray amorphous material: For comparison, the data for gaspeite, a mineral with the sum formula Ni­(CO_3_), was examined. In this mineral, the NiO_6_-octahedral units are strictly edge-linked and bridged by carbonate ions. A natural substitution with Mg is already known for this mineral.^13^ In that case, a slight distortion of the octahedral coordination environment was observed. This influences the crystal field stabilization energy (CFSE), which in turn determines the vibrations. The distortion of the octahedra causes a reduction in the CFSE, resulting in a band shift.^13^ However, the literature IR spectrum for gaspeite does not match our experimentally observed one. Furthermore, it occurs crystalline naturally as a mineral and synthetically with a known crystal structure, but the present material is X-ray amorphous. However, the compound Ni_2_(CO_3_)­(OH)_2_·*n*H_2_O, which is known to be notoriously X-ray amorphous, matches the IR spectra of the samples taken at 0 and 15 min almost perfectly.^14,15^ The vibrational spectra are highly sensitive to changes in coordination, so that a band shift from 1370 cm^–1^ (sample 0 min) to 1385 cm^–1^ (sample 15 min) can be observed (Figure 3a), due to the Mg content of the samples increasing from approximately 7% to 21%. Based on the elemental analysis of C, H, N and S (Table S2), the water content was calculated to be *n* = 2.5, resulting in the sum formula (Ni_1–*x*_Mg_*x*_)_2_(CO_3_)­(OH)_2_·2.5H_2_O for the solid precipitate. It was verified whether this “excess” water could be attributed to additional hydroxides. The solid solution hydroxide (Ni_1–*x*_Mg_*x*_)­(OH)_2_ known from the literature^16^ exhibits bands only above 3500 cm^–1^ and below 520 cm^–1^, with the exception of an OH-def. vibration at 1030 cm^–1^, which, however, would be masked by the carbonates. The presence of a ionic hydroxide would therefore be indicated by a sharp band between 3698 cm^–1^ as for Mg­(OH)_2_ (brucite) and 3635 cm^–1^ for β-Ni­(OH)_2_.^16^ In this region, however, only a very broad band from 3690 cm^–1^ to 2780 cm^–1^ is discernible in the experimental data, which is typical of water molecules, supporting the sum formula mentioned above (Figure S3.). Therefore, the assignment of the bands is proposed as follows: 3690 cm^–1^–2780 cm^–1^ OH str., 1597 cm^–1^ HOH_ads_ bend., 1385/1370 cm^–1^ antisym. CO str., 1072 cm^–1^ sym. CO str., 832 cm^–1^ out of plane def. OCO. The shift of the water bending mode compared to liquid water can be explained by water-cation interactions.

**Figure 3:**
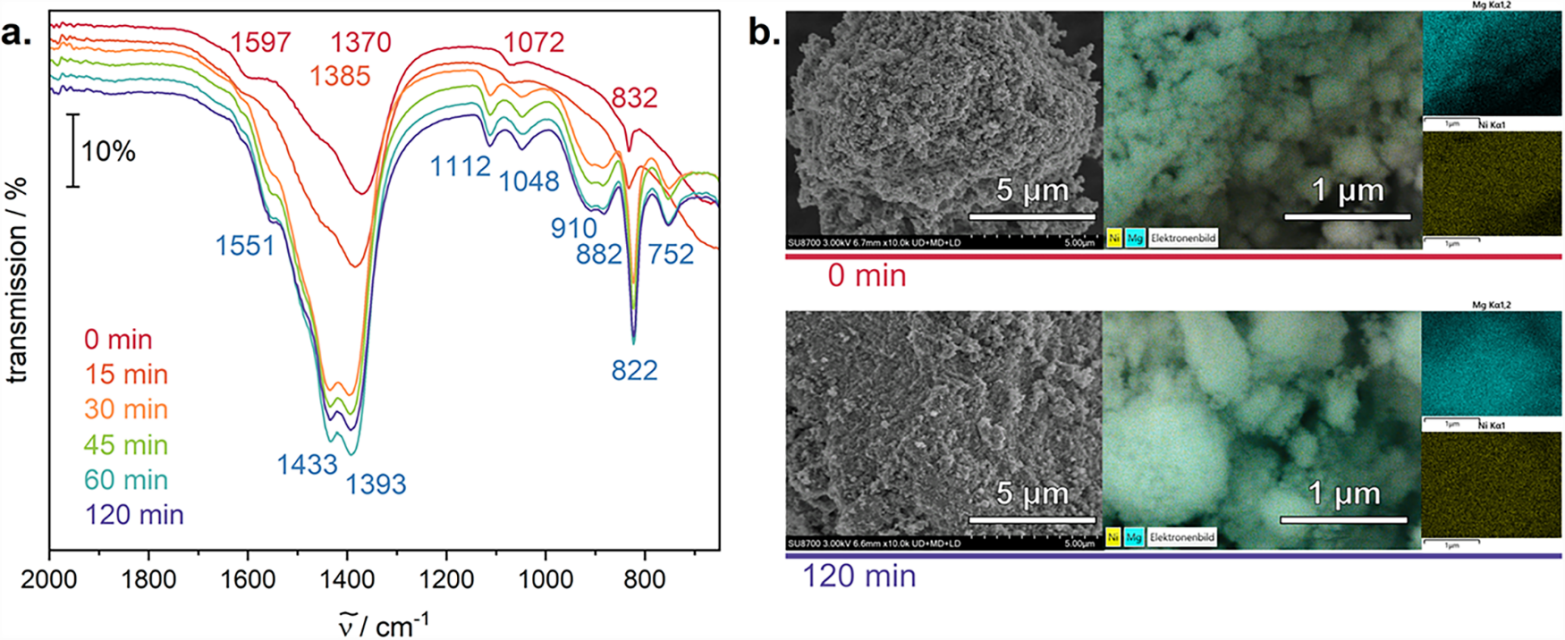
(a). magnified section of *ex situ* IR spectra of the samples (vertically offset for clarity) with increasing aging time, (b). SEM images and EDX mappings of Ni and Mg from the unaged (0 min) and the final product (120 min) as examples of the time-resolved experiment.

The IR spectra of the crystalline samples (30–120 min of aging time) show great similarity to the literature spectra for basic nickel hydroxycarbonate (Ni_12_(CO_3_)_8_(OH)_6_O·*x*H_2_O)^11^ and synthetic Hellyerite (NiCO_3_·5.5 H_2_O).^17^ For a detailed discussion, we refer the reader to the work of Rincke, Bette et al.^11,17^

The morphology of both the amorphous and crystalline samples can be described as spongy (Figures 3b, and S4). It is characterized by a highly rough surface and numerous depressions or macropores. However, upon very close inspection of the SEM images, small differences can be observed, when comparing the images of all samples side by side (Figure S4). While the amorphous particles appear to have smoother or more defined edges, the crystalline samples exhibit frayed edges on the particle borders. Additionally, the pores appear to be getting smaller with crystal growth over time. The spongy microstructure and porous texture explain the high specific surface area of 222 m^2^g^–1^ observed for the aged catalyst precursor of this composition as described in our previous work.^3^

The EDX mappings show a homogeneous distribution of the elements Ni and Mg (Figures 3b, and S5). Possible gradients in composition due to the gradual incorporation of Mg within the crystallites cannot be resolved by this method due to the small domain sizes. The different brightnesses of Ni and Mg in the map plots are caused by variations in the sample’s height and the higher scattering power of Ni. Even though it is a spatially resolved method and local variations of the composition appear naturally, the quantification of elements matches the ratios determined with ICP-OES within reasonable margin (Figure 2a). From the mappings neither particularly Mg-rich areas nor agglomerates can be observed.

### *In Situ*-IR Crystallization

To clarify whether the spontaneous crystallization is an effect of quenching or occurs in this sudden manner in the reaction as well, an *in situ* IR experiment was performed. For this experiment an equimolar ratio was chosen, which was previously shown to also crystallize in the same precursor structure,^3^ to make the effect of Mg more clearly visible. Immediately after the coprecipitation, the slurry was transferred from the glass reactor to a stirred steel reactor, which was also operated in the same synthesis station. It was sealed and the heating process was started, while pressure and temperature were monitored over a period of approximately 22 h (Figure S6). The stirring speed was kept constant at 300 rpm. During this time, IR spectra were recorded at 2 min intervals. The setup does not allow sampling under hydrothermal conditions in combination with the *in situ* IR probe. The system reached the target temperature of 103 °C after 30 min followed by a delayed pressure response (Figure 4a) with a maximum of 1.85 bar being reached after 40 min. This can be explained not only by the vapor pressure of water but with a saturation of the gas phase with CO_2_ originating from the precipitation agent CO_3_^2–^. The pressure gradually decreases again to approximately 1.2 bar within the following 6 h of the experiment. At the beginning of the experiment the general IR intensity is low, but once a certain temperature is exceeded, strong and broad bands at 1359 cm^–1^ for solvated NO_3_^–^ stretching vibrations and at 1639 cm^–1^ for the vibrational band of HOH-scissoring vibrations can be observed.^18^ The band of antisymmetric C=O stretching vibrations (∼1650 cm^–1^) of solvated HCO_3_^–^ is located in that region as well, but is in this case superimposed by water.^19–21^ These bands do not change significantly during the experiment and can therefore be assigned to the anions present in the mother liquor. With the drop from the maximum pressure after 40 min a weaker band at 1449 cm^–1^ emerges, which can be assigned to the precursors’ μ_3_-bridging carbonate stretching vibration (ν­(–C=O···M)).^18^ This is followed later by an additional band at 1410 cm^–1^ as the precursor crystallizes further (Figure 4b). The carbon dioxide is removed from the atmosphere inside the autoclave and being built in the hydroxycarbonate (Ni_1‑*y*_Mg_*y*_)_12_(CO_3_)_8_(OH)_6_O·*m*H_2_O as carbonate until equilibrium is reached. This finding is in good coherence with the IR and elemental analysis of the *ex situ* samples where the carbon content (and thus the carbonate content) is significantly higher in the crystalline than in the amorphous samples in agreement with the derived sum formulas. The pressure stabilizes at 1.12 bar after 7 h. After that time, no further changes could be observed in the spectra (Figure S6).

**Figure 4:**
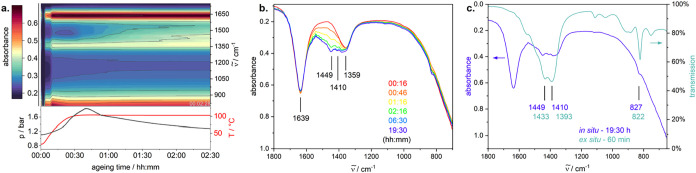
(a) Magnified section of the *in situ* IR spectra during hydrothermal aging as intensity heat map (top) with simultaneous pressure and temperature monitoring (bottom); (b) selected IR spectra of the *in situ* experiment (pictured with inverted *y*-axis for better comparison); (c) comparison of selected *in situ* and *ex situ* spectra (pictured with independent *y*-axes).

The temporal delay of crystallization compared to the *ex situ* experiment can be explained by the longer time needed to reach the target temperature. In the thick-walled steel reactor, it is reached after 30 min whereas in the smaller volume and thin-walled glass pressure reactor the temperature is reached almost instantly in the aluminum heating block. In the comparison between the *in situ* and *ex situ* spectra (Figure 4c) a shift of bands can be observed. This is caused by the different surrounding as the water molecules affect the bond strength through additional hydrogen bonding.^17^ When considering the spectra side by side, the deformation vibration band at 822 cm^–1^ (δ­(OCO)) from the *ex situ* experiment is visible as a small shoulder in the *in situ* experiment, which also intensifies as crystallization progresses. The *in situ* IR experiment has confirmed the gradual incorporation of carbonate into the crystallizing solid. This seems to coincide with the incorporation of Mg into an initially Mg-poor crystalline precursor as observed by compositional and Rietveld analysis of the *ex situ* samples. The results clearly demonstrate the necessity of hydrothermal aging, which is a key step to adjust the nickel-to-magnesium ratio in the solid catalyst precursor. This step is required to incorporate not only the full amount of Mg, but also the carbonate anions during crystallization. The latter requires a sealed reaction vessel, as the temperature (and pressure) increase needed to trigger crystallization would otherwise lead to a loss in carbonate by CO_2_ evaporation.

The quality of the finally resulting catalyst is greatly dependent on the precise adjustment of the nickel-to-magnesium ratio and thus on the hydrothermal crystallization step. Further, it is evident that thorough catalyst optimization requires also a careful setting of the reduction conditions and control over the solid-sate chemistry of metal particle formation, which is beyond the scope of this work. However, a uniform dispersion of Ni atoms and the control of their concentration in the solid is an important prerequisite for such systematic studies.

## Conclusion

From the combination of the experimental and analytical data the following equation for the reaction in aqueous solution is proposed (with *y* > *x*)­

$$6{{(\mathrm{Ni}}_{1-x}{\mathrm{Mg}}_{x})}_{2}\left({\mathrm{CO}}_{3}\right){\left(\mathrm{OH}\right)}_{2}+2{{\mathrm{HCO}}_{3}}^{-}+\left(y-x\right){\mathrm{Mg}}^{2+}\to {{(\mathrm{Ni}}_{1-y}{\mathrm{Mg}}_{y})}_{12}{{\left({\mathrm{CO}}_{3}\right)}_{8}\left(\mathrm{OH}\right)}_{6}O+3H_{2}O+2{\mathrm{OH}}^{-}$$

From the *ex situ* IR spectra and the elemental analysis the sum formula of the amorphous precipitate ((Ni_1–*x*_Mg_*x*_)_2_(CO_3_)­(OH)_2_·*n*H_2_O) could be determined. At a basic pH, HCO_3_^–^ ions are present in the solution due to the carbonate-carbonic acid equilibrium. The drop in pressure indicates the formation of a solid which has a higher stoichiometric carbonate number relative to the precipitate. The onset of crystallization from the precipitate to the crystalline compound occurs under 30 min after reaching target temperature. After this the Mg content is increasing slowly and only crystal growth takes place. The formation of the structure ((Ni_1‑*y*_Mg_*y*_)_12_(CO_3_)_8_(OH)_6_O·*m*H_2_O) was confirmed by X-ray diffraction (*ex situ*) as well as IR spectroscopy (*ex situ* and *in situ*). The ratio of the metal ions Ni^2+^ and Mg^2+^ is not predetermined for the target compound within the precipitate but evolves over the reaction indicated by a shift of IR bands, ICP-OES and EDX data as well as Rietveld refinement (*ex situ*). The complementarity and overlap between the various *ex situ* and *in situ* methods successfully advanced our understanding of the core processes involved in the wet chemical synthesis of a precursor for a highly active methanation catalyst. The hydrothermal aging step has proven to be not only a direct crystallization but an integral part of the synthesis, as it alters both the chemical composition and the crystal structure, and this only being achievable in a closed system.

## Supplementary Material



## Data Availability

Data Availability Statement: The data that support the findings of this study are available from the corresponding author upon reasonable request.

## Notes

Additional experimental results, details, graphs, and images (PDF)
