# Supplementary material for: Investigating the Crystallization of a Hydroxycarbonate Precursor for a Ni–Mg–Catalyst through In Situ and Ex Situ Methods
Source: Inorg Chem. 2026 Jul 23;65(30):17552–7. doi: 10.1021/acs.inorgchem.6c02129 (PMC13439655; doi:10.1021/acs.inorgchem.6c02129)
Supplement: Supplementary file 1 [file ic6c02129_si_001.pdf]

Supporting Information

Title: Investigating the Crystallization of a Hydroxycarbonate Precursor for a Ni-Mg-Catalyst through In situ and Ex situ Methods

Authors: Anna Wolf,[a] Sebastian Mangelsen[a] and Malte Behrens*[a]

[a] Institute of Inorganic Chemistry and Kiel Nano Surface and Interface Science (KiNSIS)
Kiel University,
Max-Eyth-Str. 2, 24118 Kiel, Germany

*E-Mail: mbehrens@ac.uni-kiel.de

The authors have cited additional references within the Supporting Information.

Additional Results

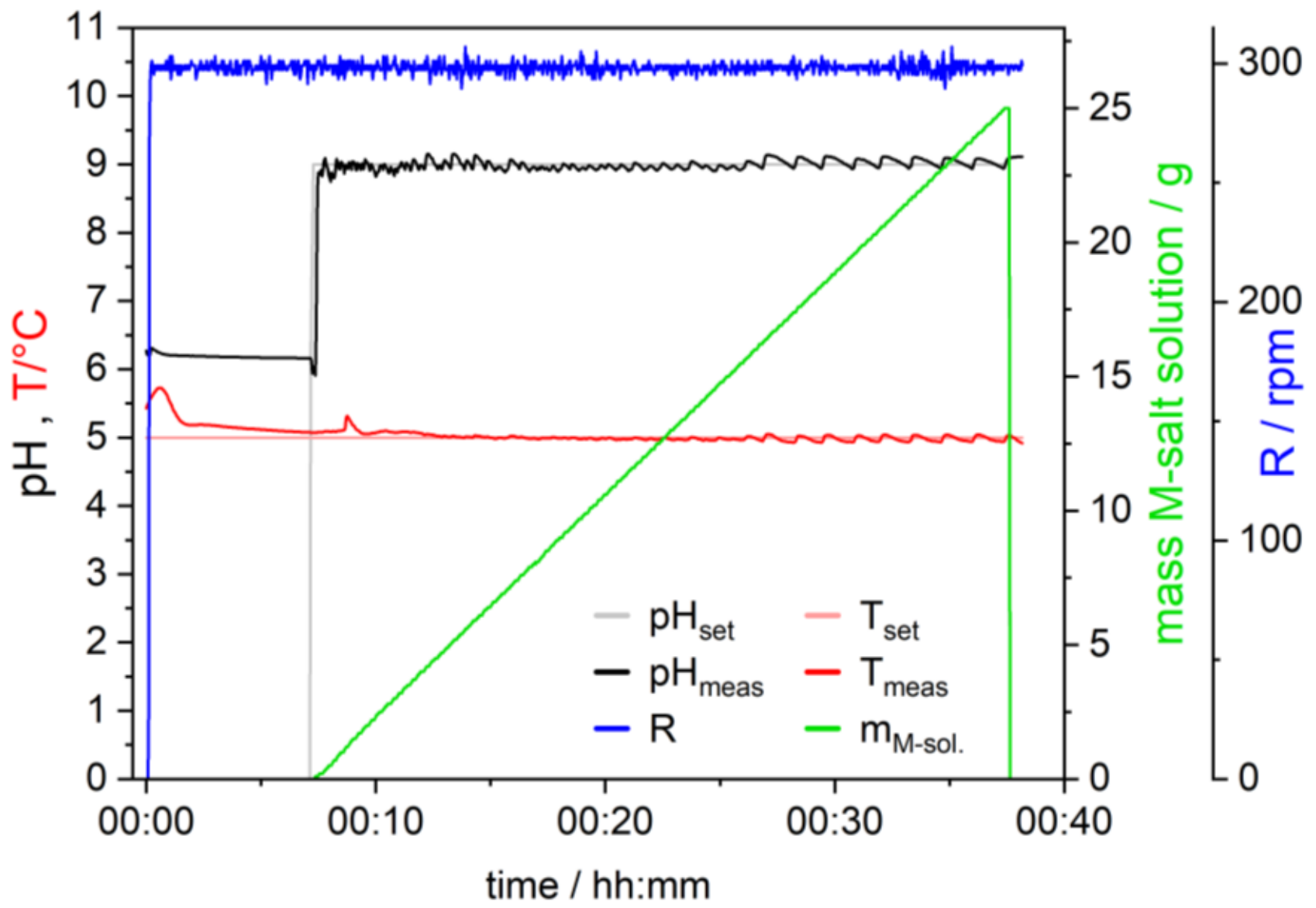

Figure S1: Synthesis protocol of the coprecipitation before the transfer to the Pyrex vials.

Table S1: Results of the Rietveld refinements.

Sample	Lattice parameter *a* / Å	Mg occupancy	Avarage diameter / nm	GoF	r_wp
**30 min**	8.4106(6)	0.15(1)	3.5(2)	1.27	2.23
**45 min**	8.4115(4)	0.19(1)	3.8(2)	1.21	2.27
**60 min**	8.4136(4)	0.22(1)	3.8(2)	1.25	2.44
**120 min**	8.4144(4)	0.21(1)	4.2(2)	1.18	2.31

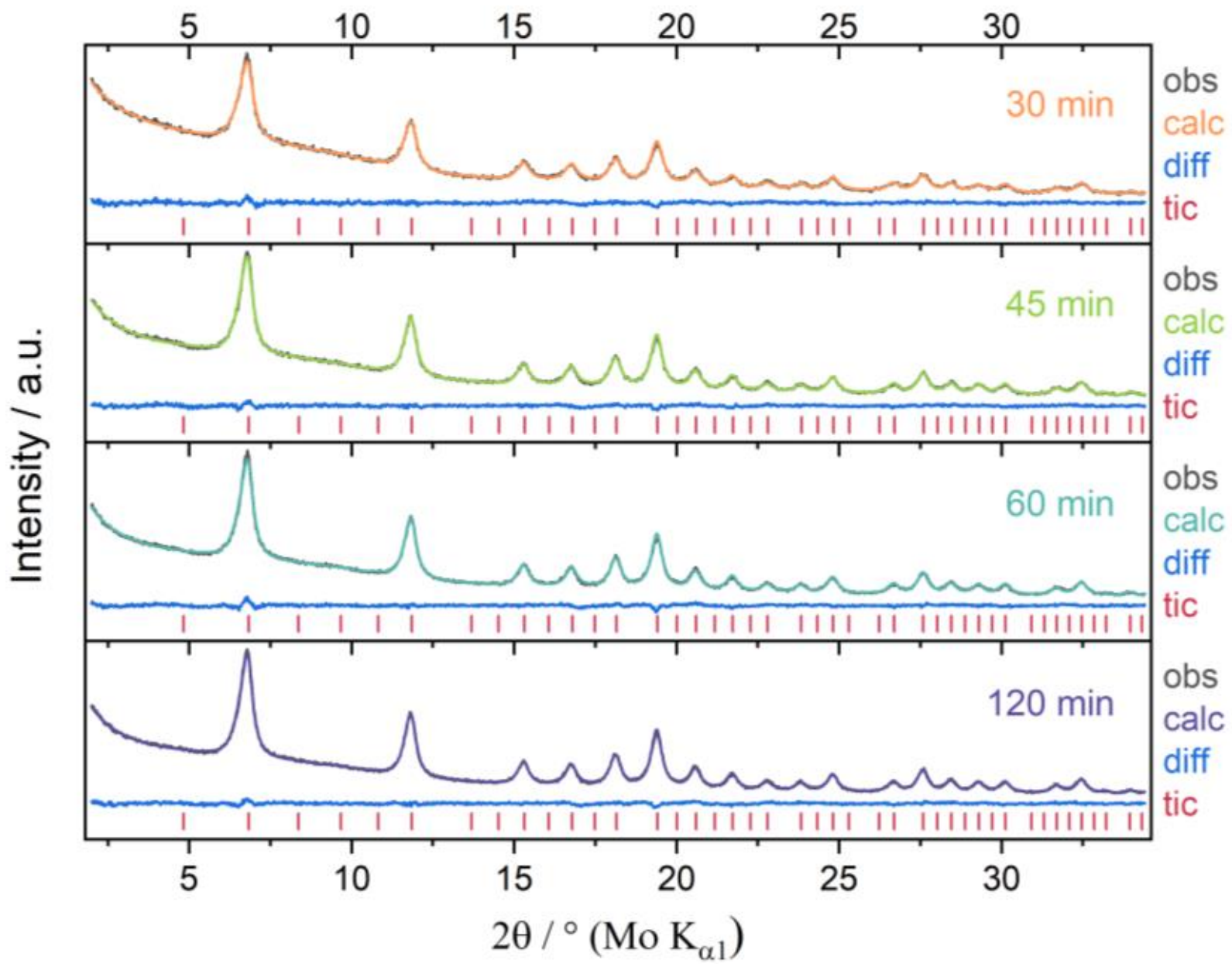

Figure S2: Difference plots of the Rietveld refinements of the crystalline samples. Pictured are the observed experimental data (*obs* – grey) overlaid by the calculated function (*calc* – various) and their difference plot (*diff* – blue). The allowed peak positions for modified $(Ni_{1-x}Mg_x)_{12}(CO_3)_8(OH)_6O \cdot y\ H_2O$ with altered parameters are marked (*tic* – red).

Table S2: Results of the elemental analysis of the elements C, H, N and S from the samples taken at different aging times, as well as theoretical compositions.

	**Sample**	**N %**	**C %**	**H %**	**S %**	**Weight/mg**
**Measured values**	0 min	0	4.66	2.733	0	4.121
	15 min	0.01	5.24	2.771	0	3.719
	30 min	0.01	7.13	1.984	0	2.712
	45 min	0.01	7.65	1.826	0	3.512
	60 min	0.02	7.67	1.844	0	2.553
	120 min	0.02	7.61	1.77	0	2.984
**Theoretical values**	$(Ni_{0.927}Mg_{0.073})_2(CO_3)(OH)_2$ * 2.5 H_2O	-	4.78	2.81	-	-
	$(Ni_{0.79}Mg_{0.21})_2(CO_3)(OH)_2$ * 2.5 H_2O	-	4.96	2.92	-	-

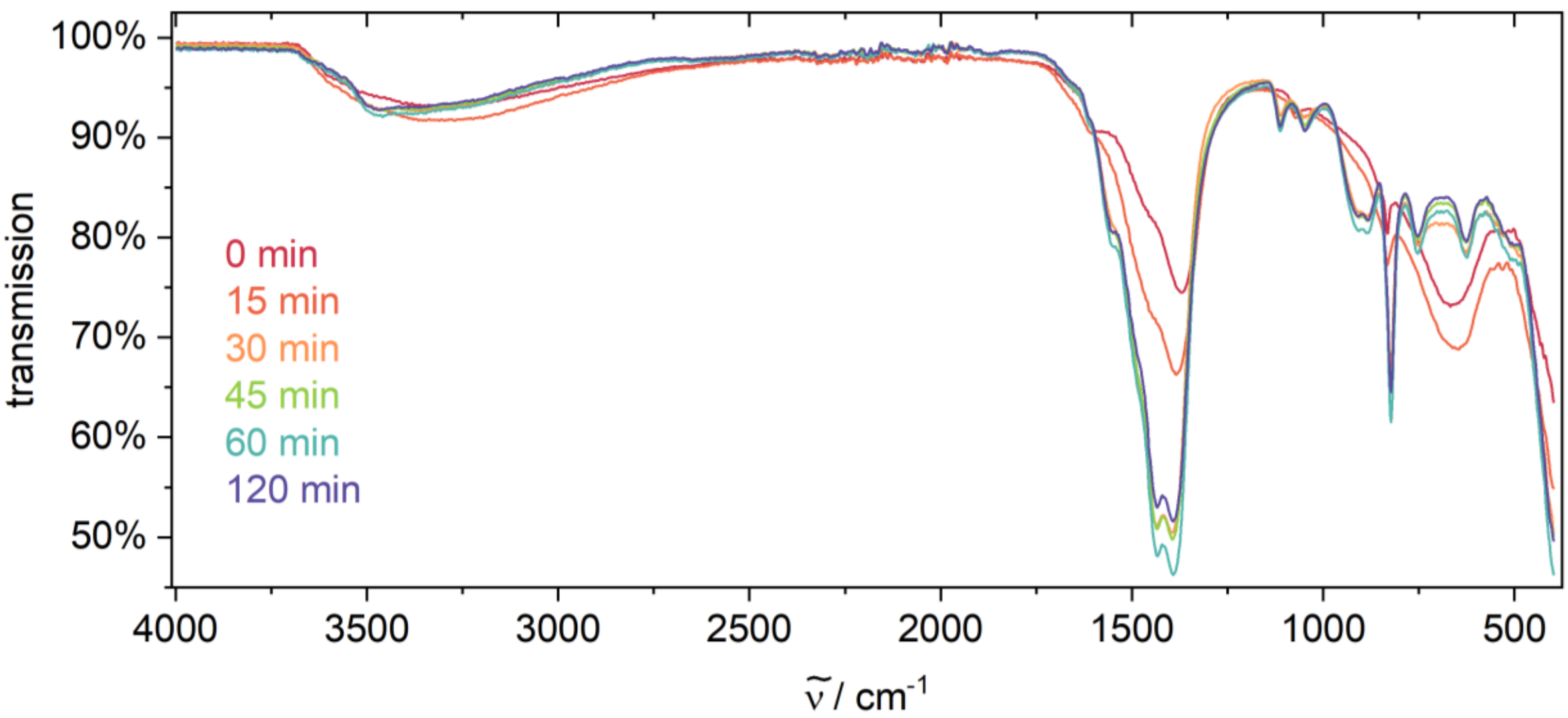

Figure S3: Complete section of *ex situ* IR spectra of the samples with increasing aging time.

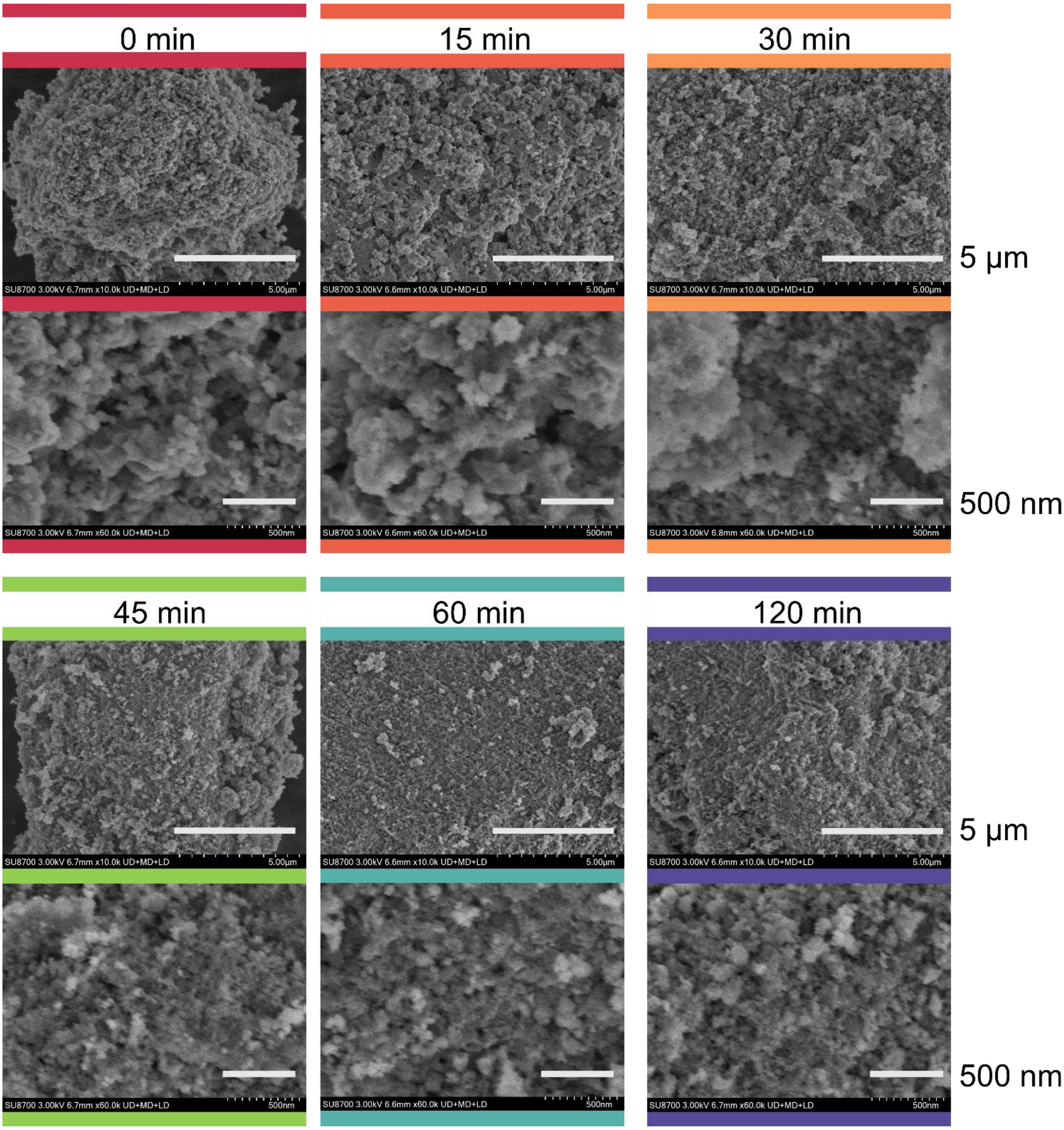

Figure S4: SEM images of *ex situ* sample series of different aging times with 10,000x (*top*) and 60,000x (*bottom*) magnification.

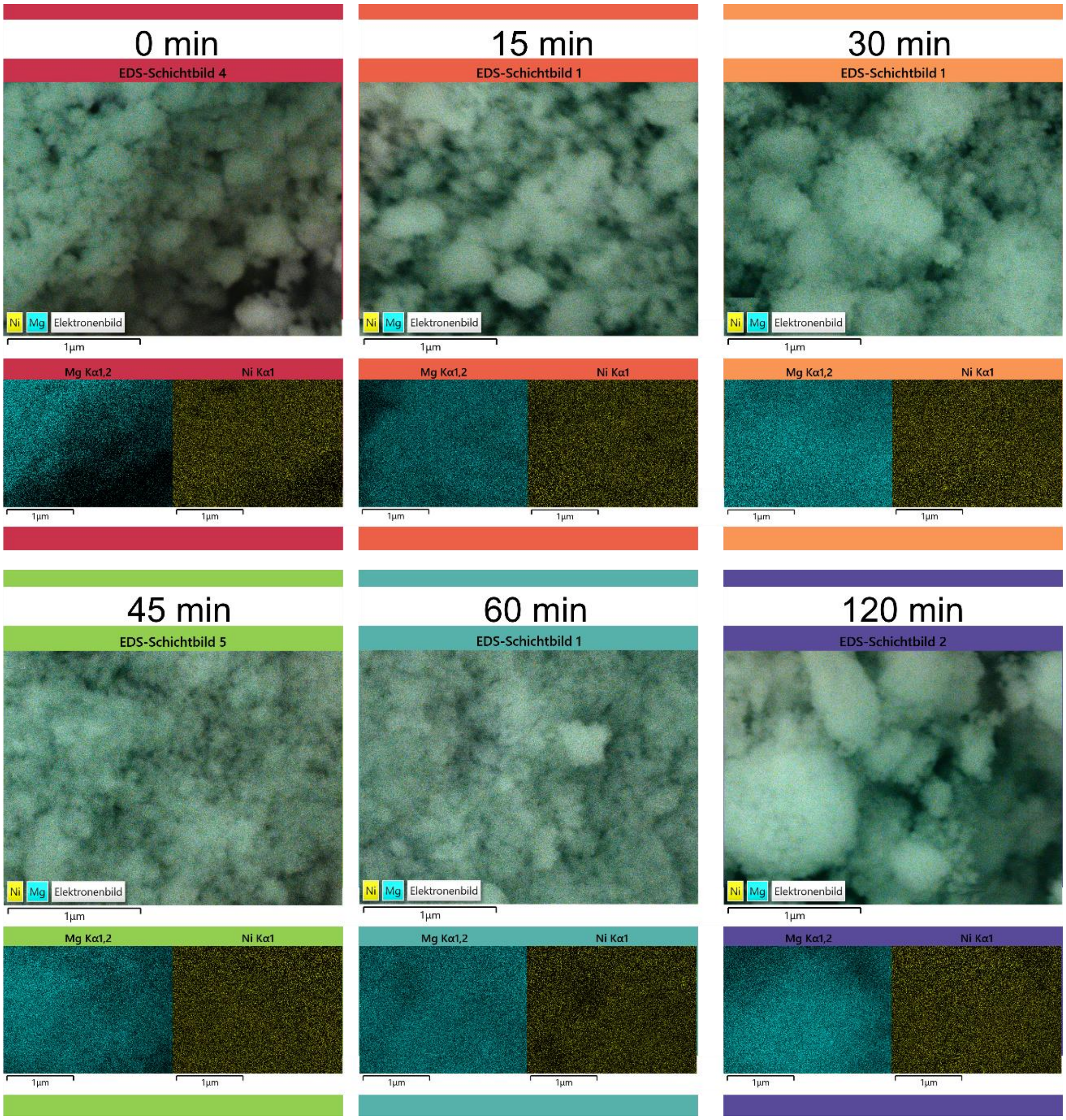

Figure S5: EDX mapping of *ex situ* sample series of different aging times with overlay (*top*) and single type atom (*bottom*) images. Ni – yellow, Mg – turquoise.

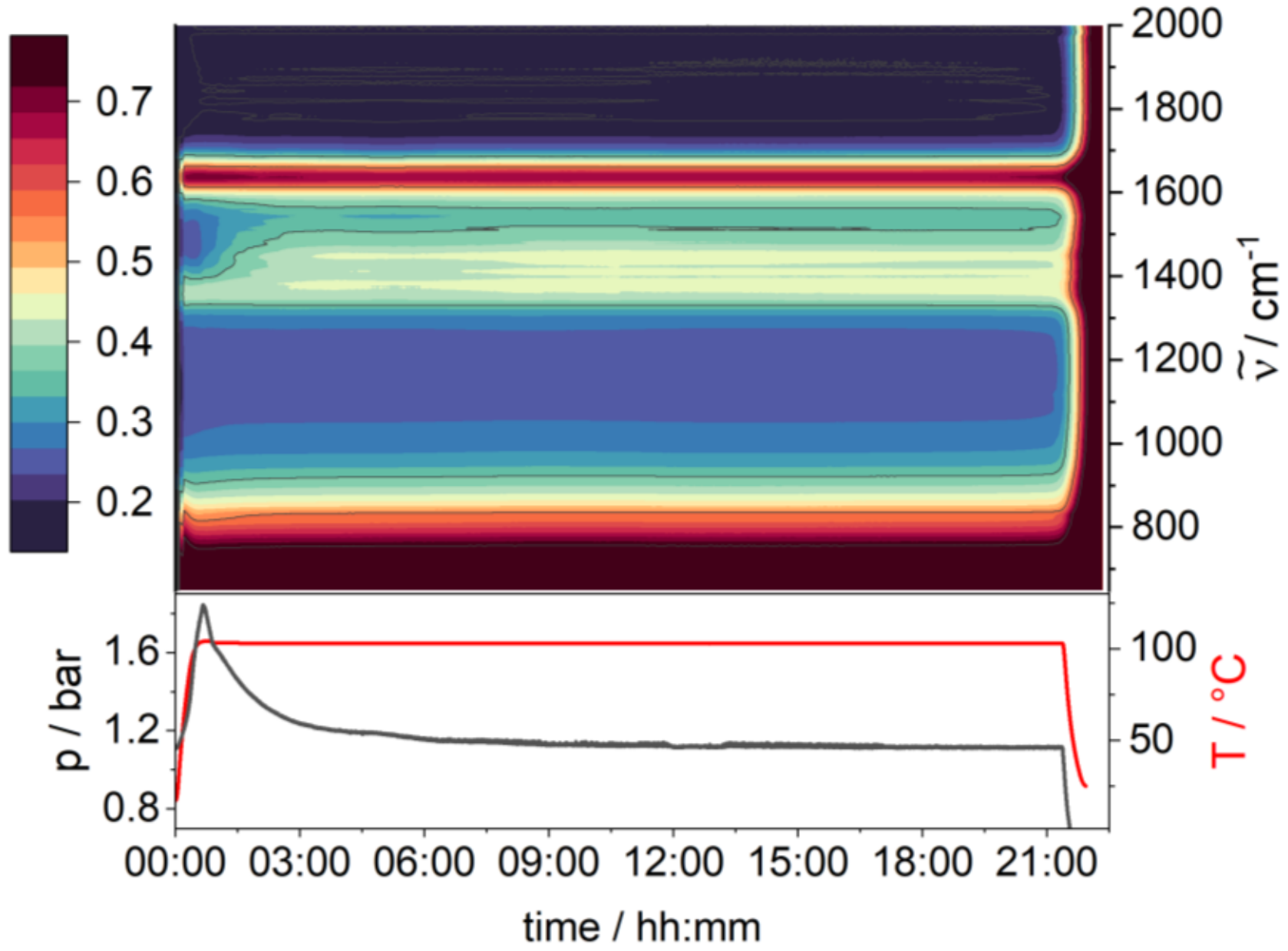

Figure S6: Complete section of the *in situ* IR spectra during hydrothermal aging as intensity heat map (top) with simultaneous pressure and temperature monitoring (bottom).

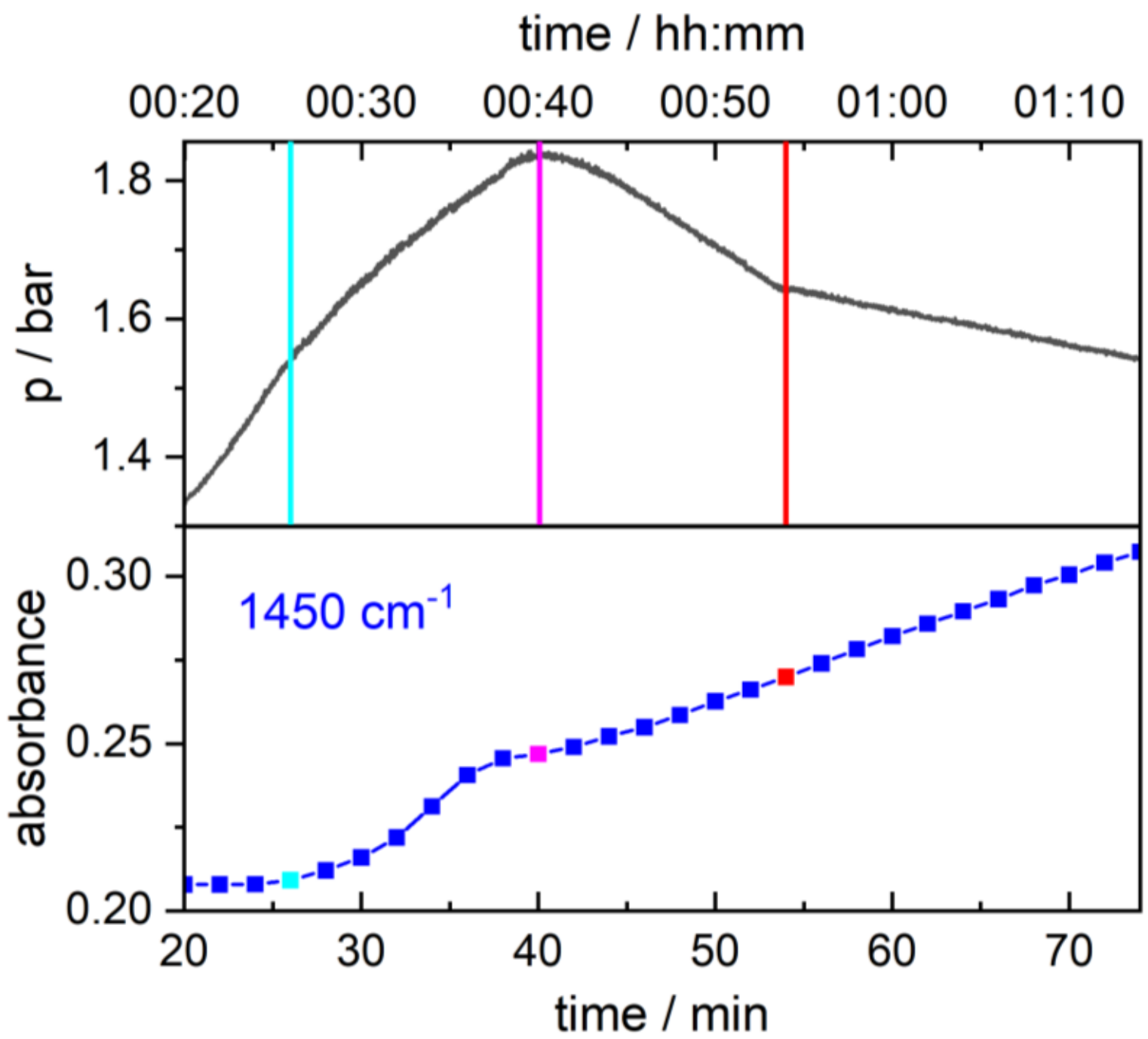

Figure S7: Magnified section of the pressure monitoring and the intensity of the band at 1450 cm^{-1} in the *in situ* IR spectra during hydrothermal aging time. Marked events: slope change of pressure (*turquoise*), maximum pressure (*pink*) and second slope change (*red*).
